# Micron-scale mapping of megagauss magnetic fields using optical polarimetry to probe hot electron transport in petawatt-class laser-solid interactions

**DOI:** 10.1038/s41598-017-08619-1

**Published:** 2017-08-21

**Authors:** Gourab Chatterjee, Prashant Kumar Singh, A. P. L. Robinson, D. Blackman, N. Booth, O. Culfa, R. J. Dance, L. A. Gizzi, R. J. Gray, J. S. Green, P. Koester, G. Ravindra Kumar, L. Labate, Amit D. Lad, K. L. Lancaster, J. Pasley, N. C. Woolsey, P. P. Rajeev

**Affiliations:** 1 Tata Institute of Fundamental Research, Homi Bhabha Road, Mumbai, 400005 India; 2 Central Laser Facility, STFC Rutherford Appleton Laboratory, Chilton, Didcot OX11 0QX UK; 3 York Plasma Institute, University of York, Heslington, York YO10 5DQ UK; 4 SUPA, Dept. of Physics, University of Strathclyde, Glasgow, G4 0NG UK; 5 Intense Laser Irradiation Laboratory (ILIL), INO-CNR Pisa, Italy; 6 Present Address: Max Planck Institute for the Structure and Dynamics of Matter, Luruper Chaussee, 22761 Hamburg Germany

## Abstract

The transport of hot, relativistic electrons produced by the interaction of an intense petawatt laser pulse with a solid has garnered interest due to its potential application in the development of innovative x-ray sources and ion-acceleration schemes. We report on spatially and temporally resolved measurements of megagauss magnetic fields at the rear of a 50-*μ*m thick plastic target, irradiated by a multi-picosecond petawatt laser pulse at an incident intensity of ~10^20^ W/cm^2^. The pump-probe polarimetric measurements with micron-scale spatial resolution reveal the dynamics of the magnetic fields generated by the hot electron distribution at the target rear. An annular magnetic field profile was observed ~5 ps after the interaction, indicating a relatively smooth hot electron distribution at the rear-side of the plastic target. This is contrary to previous time-integrated measurements, which infer that such targets will produce highly structured hot electron transport. We measured large-scale filamentation of the hot electron distribution at the target rear only at later time-scales of ~10 ps, resulting in a commensurate large-scale filamentation of the magnetic field profile. Three-dimensional hybrid simulations corroborate our experimental observations and demonstrate a beam-like hot electron transport at initial time-scales that may be attributed to the local resistivity profile at the target rear.

## Introduction

An intense laser pulse focussed on a solid target can generate relativistic electron currents reaching mega-ampere levels^1–3^. The generation and transport of these hot electron currents through the solid are central to a number of potential applications including the development of novel x-ray sources^4^ and alternate particle acceleration schemes^5^. For instance, the hot electron distribution at the target rear seeds the growth of the sheath fields responsible for target-normal-sheath-acceleration and plays an important role in determining the laminarity and spatial uniformity of the proton and ion emission profiles^6^ – a principal parameter in deciding the viability of such intense-laser-based energetic ion sources for their diverse applications in medical imaging and ion therapy^5^. In addition, in the fast ignition variant of inertial confinement fusion, the generation and collimated transport of the ignitor electron pulse is crucial to the energy transfer from the point of laser-coupling to the imploded fuel hot-spot. Self-divergence of the hot electron beam results in inefficient heating of the fuel, imposing impractical demands on the ignitor laser pulse in terms of energy and intensity^3^. Consequently, various experimental techniques^7–12^ corroborated by numerical simulations^13–16^ have been designed to investigate hot electron transport by employing a variety of diagnostics such as rear-side optical self-emission^7, 10^, and proton radiography^8, 12^. The hot electron transport process is inherently transient and confined to micron-scales, and therefore warrants diagnostics with a *simultaneous spatio-temporal resolution* capable of capturing the rapidly evolving dynamics of the hot electron distribution^17^.

Mapping the evolution of the magnetic fields produced by the hot electron currents streaming into the solid target can open a window to the complex dynamics of the hot electron currents in the solid target. These magnetic fields, with magnitudes approaching gigagauss levels^18, 19^, are pivotal in determining the propagation of the hot electron currents that generate them, leading to a complex interplay between the hot electron currents and the magnetic fields.

In order to study the influence of the target material in the hot electron transport process, one needs to measure the magnetic fields set up at the rear surface of the target^20^. The spatial profile of the magnetic fields at the target rear is determined by the spatial profile of the hot electron currents evolved through their transport across the bulk of the target. Detailed measurements of these magnetic fields enable one to make detailed inferences about transport in the bulk target as well as detailed comparisons with numerical models. To understand the dynamics of hot electron transport, a simultaneous spatio-temporal characterisation of the magnetic fields at the target rear is therefore required.

Previous magnetic field measurements have mostly been limited to the laser-irradiated front surface of the target. For instance, the X-wave cutoff of laser-generated harmonics^21^ provides a measure of the magnetic fields at the critical surface at the target front. Faraday rotation of an external probe^22, 23^, provides temporal snapshots of the magnetic fields in the underdense plasma, albeit integrated along the transverse density profile. Proton deflectometry can also provide time-resolved magnetic field measurements^17^, albeit, in principle, integrated through the thickness of the target. Although studying the proton beam profile at various energies can shed light on the profile of the hot electron distribution at different instants of time, the temporal resolution of this technique may be limited by the duration of the proton pulse and its time of flight along with the energy resolution in its measurement.

Here, we present an optical polarimetry technique that can be a complementary diagnostic, providing spatially and temporally resolved snapshots of hot electron transport through solids. We employ a time-delayed optical probe, reflected off the critical surface at the target rear, for the simultaneous spatial and temporal mapping of the magnetic fields at the target rear. The magnetic fields induce a change in the polarization state of the probe beam. Since the magnetic fields are mostly azimuthal in nature, they induce an ellipticity in the reflected probe due to the Cotton-Mouton effect^24–26^. The polarimetric measurements, localized at the target rear and spatially resolved along the transverse plane, map the picosecond-scale temporal evolution of the magnetic fields at the target rear, produced by the hot electron transport through the target. Consequently, this technique provides a generic recipe of magnetic field measurement, influenced only by the hot electron distribution at the target rear, and in principle with a spatial resolution decided by the diffraction-limited optical resolution of the probe imaging setup, and a temporal resolution limited by the laser pulsewidth.

This paper presents the first spatio-temporally resolved magnetic field measurements at the target rear for a multi-picosecond, petawatt driver laser pulse using optical pump-probe Cotton-Mouton polarimetry, yielding new insights into the principal characteristics of hot electron transport through solid targets under fast-ignition relevant irradiation conditions. We observed signatures of a relatively smooth hot electron transport in 50-*μ*m thick plastic (CH) targets until ~5 ps after the incidence of the main interaction pulse, mirrored in the annular magnetic field profile measured at the target rear. At later time-scales of ~10 ps, however, a diffused and filamented magnetic field profile was observed. At these time-scales, the dynamics in the plasma sheath at the rear of the target^27, 28^, including refluxing^12^, influence the measurements, along with the filamentation in the hot electron distribution^1^. Most notably, our experimental observations identify an initial regime where the transport is relatively smooth in a material that is non-conducting at room temperature. This conforms to the proton radiography measurements by Quinn *et al*.^12^, but is contrary to the generic description of such targets typically associated with a highly structured hot electron transport, as opposed to metals that are characterized by a smooth hot electron beam profile^8–10^, inferred from time-integrated measurements. Our experiments aim at resolving this apparent contradiction by a spatio-temporally resolved study of hot electron transport through solids and are supported by the results of three-dimensional (3D) hybrid simulations that elucidate the dynamic role played by the transient temperature-dependent local resistivity profile of the target.

## Results

**Figure 1:**
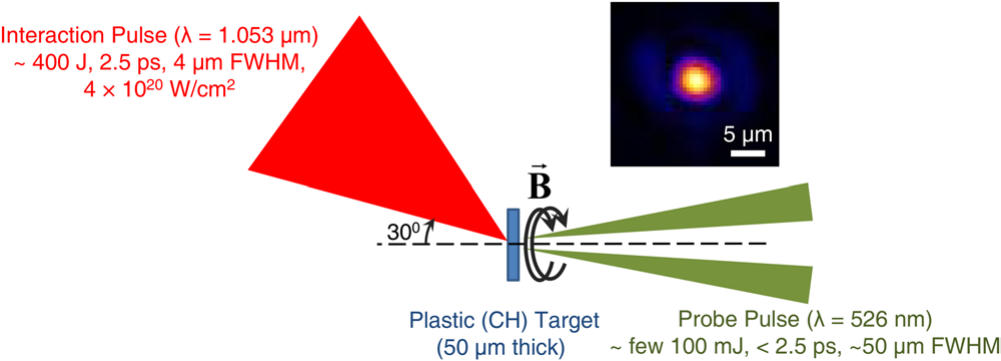
Schematic of the experimental setup, showing the main interaction pulse generating the magnetic fields (B) in the plastic (CH) target, which are probed at the target rear by a time-delayed probe pulse. The inset shows the typical transverse profile of the focal spot of the main interaction pulse on the target.

The experiment was performed at the Rutherford Appleton Laboratory using the Vulcan Petawatt laser, delivering more than 400 J on target at a central wavelength of 1.053 *μ*m over a pulse duration of 2.5 ps and at an irradiance of ~4 × 10^20^ W/cm^2^. A schematic of the experimental setup is shown in Fig. 1. The magnetic fields were inferred from a pump-probe polarimetric diagnostic^24–26^, employing a linearly-polarized, time-delayed and frequency-doubled (*λ* = 526 nm) probe pulse, derived from the main interaction pulse, and focused to the rear of the target at near-normal incidence. The polarimetric measurements indicated that the predominant polarization change consisted of an induced ellipticity in the probe due to the azimuthal nature of the self-generated magnetic fields according to the Cotton-Mouton effect. The Faraday rotation of the normally incident probe due to any axial component of the magnetic field was found to be below the threshold of detection.

**Figure 2:**
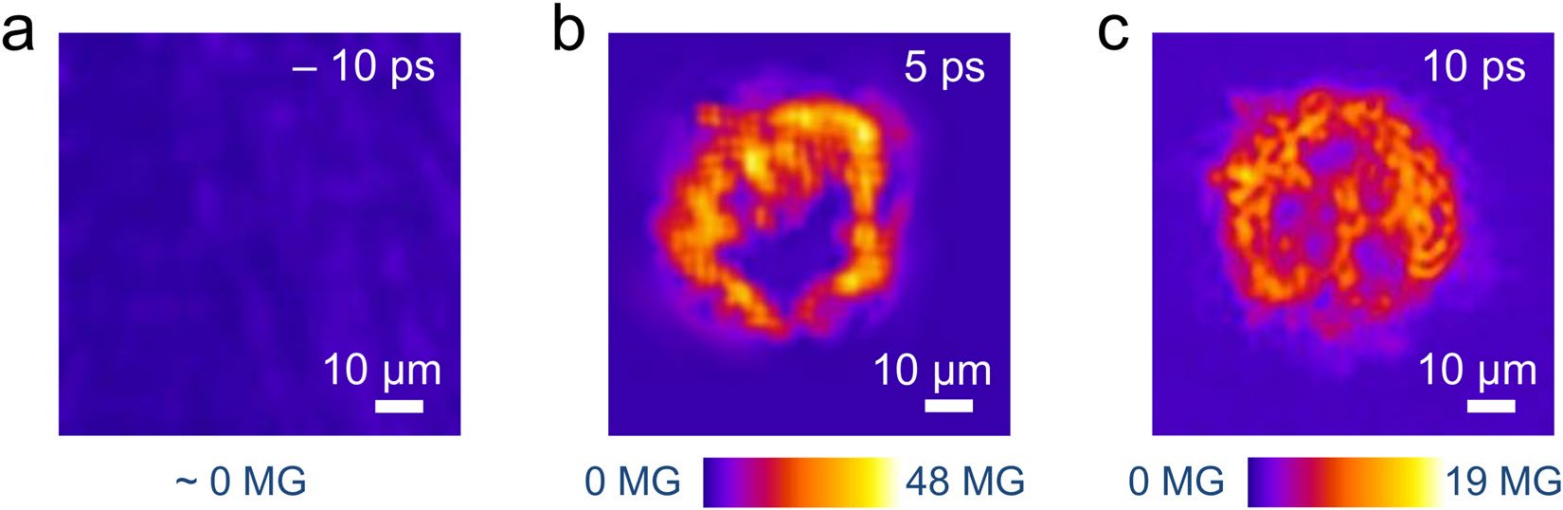
The magnetic field profile at the rear of a 1 mm × 1 mm, 50-*μ*m thick plastic (CH) target at a temporal delay of (**a**) - 10 ps, (**b**) 5 ps and (**c**) 10 ps after the main interaction pulse (negative delay indicates that the probe reached the target before the main interaction pulse). The spatial resolution of the optical imaging setup was <10 *μ*m and the error in the estimation of the peak magnetic field is ±5 MG. A null magnetic field profile at negative time-delays defines the noise level of the measurement and serves as a ‘control’ measurement. An annular magnetic field profile can be observed at 5 ps, indicating a relatively smooth hot electron distribution. However, at 10 ps, a large-scale filamentation of the hot electron distribution at the target rear can be observed.

Figure 2 presents the magnetic field profiles at the rear of a 50-*μ*m thick CH target at different instants of time. At a negative time delay of 10 ps (that is, for the probe reaching the target 10 ps before the main interaction pulse), a null magnetic field profile was obtained (Fig. 2a), indistinguishable from the background. This measurement defines the noise level for the magnetic field and shows that the probe as well as the prepulse does not induce any perturbative effects on the magnetic field measurements reported here.

Figure 2b and c show the magnetic field profiles at 5 ps and 10 ps after the main interaction pulse respectively. The magnetic field reaches local peak values of ~50 MG at a temporal delay of 5 ps. The most significant feature in the profile, however, is the annular distribution of the magnetic field with a central hollow (Fig. 2b), observed repeatedly in our experiments. Qualitatively similar magnetic field profiles with a central hollow were also observed for 100-*μ*m thick CH targets at 5 ps. Such an annular magnetic field profile at the target rear^13–15^, is indicative of a beam-like distribution of the hot electrons exiting the target^29^. In contrast, the magnetic field profile 10 ps after the main interaction pulse (Fig. 2c) shows a diffused magnetic field profile with pronounced filamentation. At these time-scales, the hot electron distribution can be filamented inside a CH target^1, 2, 8–10^, which can be further influenced by the dynamics in the rear-side sheath^12, 27, 28^.

The magnetic fields observed at the target rear are generated in conjunction with the sheath field, which is set up when the hot electron beam generated at the target front impinges on the rear surface of the target^20^. As the hot electron beam has a finite transverse extent, so will the sheath field at early times. This leads to a significant net ∇ × **E**, which generates a large magnetic field (∂**B**/∂*t*), an analysis of which is given in ref. 30. A simplistic order-of-magnitude estimate of the magnitude of these fields identifies their mechanism of generation, as follows. Typically, *B*~*E*_*sheath*_/*c*, and as *E*_*sheath*_ is of the order of a few TV/m^5^ we see that *B* > 10 MG, which agrees with the magnitude of the magnetic fields observed in our experiments.

Detailed simulations were carried out using ZEPHYROS^31, 32^, a 3D hybrid code, to probe the hot electron transport process in the CH target. The simulations assume that the energy is deposited into the target and investigate the propagation of the hot electrons, following the energy deposition. The measured magnetic fields are established when the sheath field first forms at the target rear surface, which occurs following the first transit of the hot electrons through the target. Consequently, early time-scales are the most significant, and hence running the simulations up to 0.7 ps was found to capture most of the relevant physics (given magnetic diffusion time-scales are significantly longer than the experimental time-scales for our experimental parameters). The qualitative outcome of the simulations was found to be quite robust and independent of varying the energy deposition time or the initial angular divergence of the hot electron beam.

**Figure 3:**
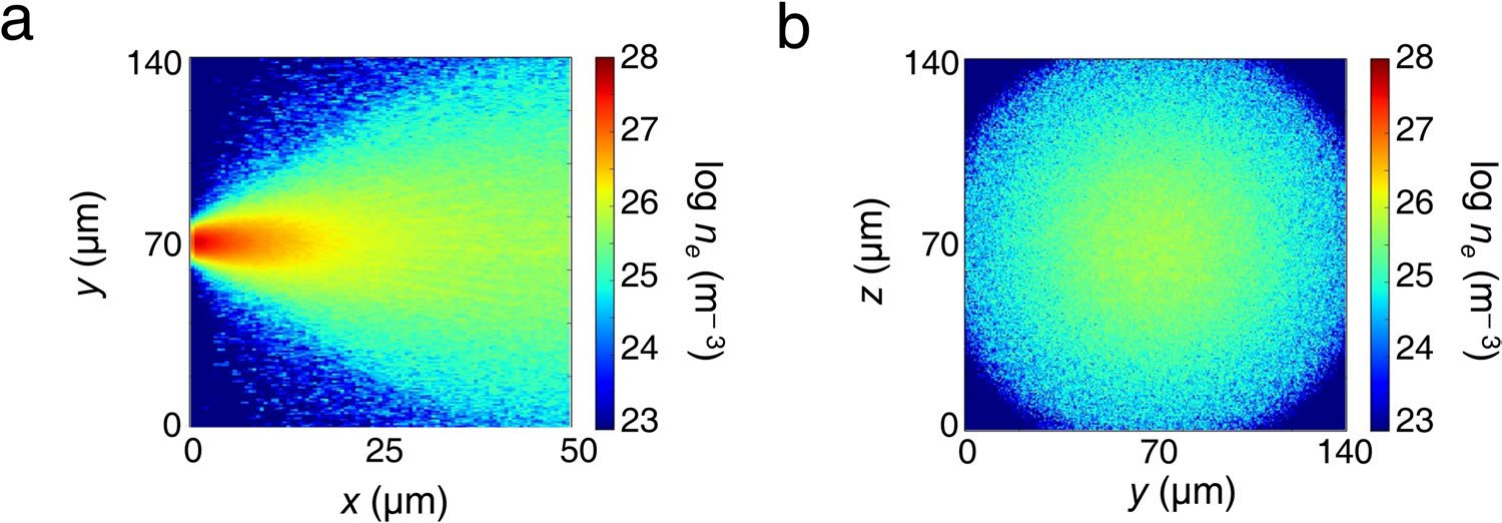
Results of the ZEPHYROS simulations at 0.7 ps, showing the (**a**) longitudinal and (**b**) transverse profiles of the electron density *n*_*e*_. These simulations corroborate the experimentally observed collimated hot electron transport pattern through the target at early time-scales.

The results of the simulations are shown in Fig. 3, where Fig. 3a and b give the longitudinal and transverse snapshots of the electron distribution 0.7 ps after the interaction, as the electrons propagate through the target. It is clear that, although the electrons diverge as they pass through the target, the distribution remains beam-like at initial time-scales, as shown in Fig. 3a. The transverse hot electron density profile at the rear surface (Fig. 3b) illustrates this clearly, indicating a relatively smooth hot electron distribution. Such a hot electron distribution should give rise to an annular magnetic field profile^29^. Had the beam fragmented into several beamlets due to resistive filamentation^1^ in the bulk of the target, a more complex, fragmented magnetic field profile would have emerged. It is therefore reasonable to interpret our experimental results in Fig. 2 as providing evidence of minimal fragmentation at early time-scales, corresponding to a beam-like hot electron distribution and an annular magnetic field profile. In contrast, later time-scales are characterized by a fragmented magnetic field profile due to the onset of large-scale filamentation, further affected by the sheath-field dynamics at the target rear^12, 27, 28^.

## Discussion

Hot electron distribution at the rear of a solid target is rather well-documented in literature^1, 2, 7–10, 12–16, 33–35^; targets that are non-conducting at room temperature (such as CH and glass) are typically associated with a highly filamented hot electron transport, as opposed to the rather smooth beam-like hot electron propagation in metals, supported by various *time-integrated* measurements^8, 10, 34^. Fuchs *et al*.^8^ reported on the smooth proton distribution obtained on a radiochromic film by the illumination of a ~50-*μ*m thick gold (Au) foil with an intense laser (~2 × 10^19^ W/cm^2^, 350 fs), in stark contrast with the “proton caustics” and filaments observed for polished glass (SiO_2_) targets or alternatively, CH targets or layered Au + CH targets of thicknesses varying in the range (50–100) *μ*m. Similar distinctions were also made by Manclossi *et al*.^10^, who contrasted the smooth optical emission from the rear of aluminum (Al) targets with the highly filamented emission from the CH targets, with thicknesses ranging from 10 to 100 *μ*m, when irradiated with 40 fs pulses at an incident intensity of 6 × 10^19^ W/cm^2^. However, Quinn *et al*.^12^ recently quantified the non-uniformity in the spatial profile of the proton beam emitted from the rear of Al as well as CH and SiO_2_ targets as a function of the target thickness varying between 50 and 1200 *μ*m, under irradiation conditions similar to those in our experiment (5 × 10^20^ W/cm^2^, 1 ps). While the non-uniformity parameter was found to increase significantly with increasing thickness for the CH and SiO_2_ targets, it was found to be fairly similar for 50-*μ*m thick CH and Al targets. This is a clear departure from the previously established understanding that hot electron transport in metals (such as Al) is less prone to filamentation, compared to materials such as CH. It is questionable whether this material dependence should be universal, as the details of the interaction should also depend on local target conditions, most notably the local resistivity profile. This naturally elicits an extensive spatio-temporally resolved investigation. Our experimental inferences that the hot electron distribution remains relatively smooth and almost beam-like even in a CH target for at least a few picoseconds following the incidence of the main interaction pulse are in broad agreement with the experimental results of Quinn *et al*.^12^. For a better understanding of the hot electron transport process, additional simulations were carried out, comparing the resistivity of CH with that of Al – an excellent conductor – as a function of the temperature, which is the key dynamic parameter deciding the local resistivity profile.

**Figure 4:**
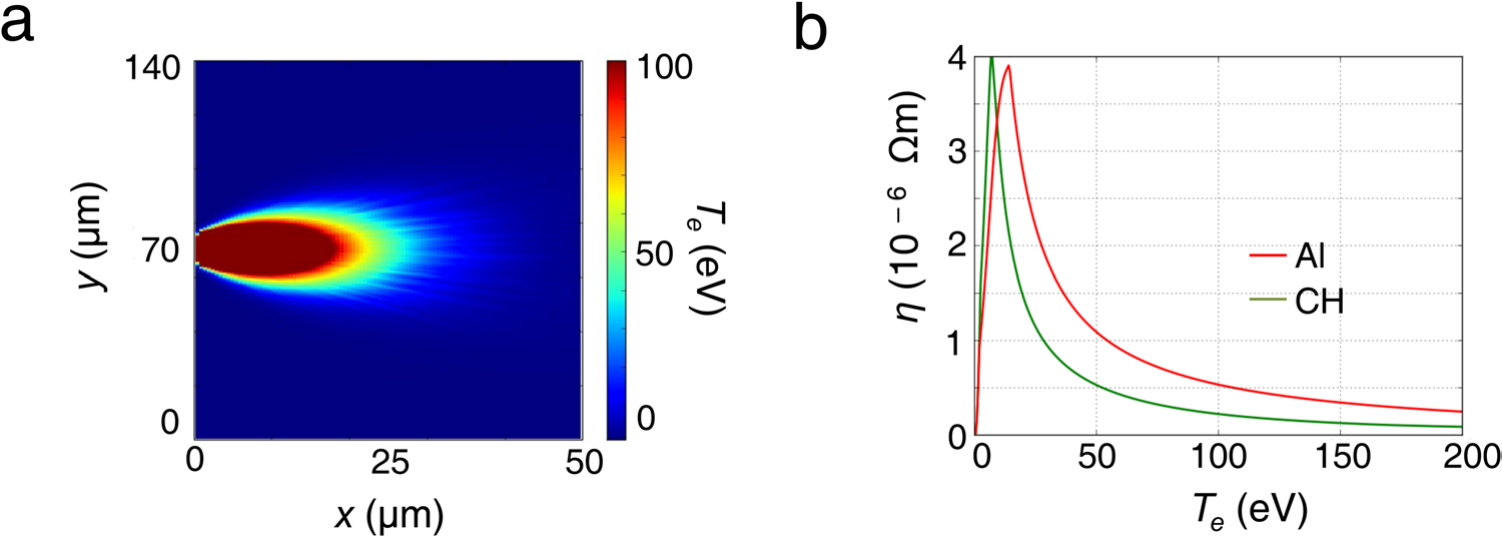
(**a**) Temperature (*T*_*e*_) contours in the *x* − *y* plane along the longitudinal direction in the CH target, 0.7 ps after the interaction. (**b**) Resistivities of CH (green) and Al (red) as a function of bulk temperature. The saturated region indicates temperatures greater than 100 eV. The simulations indicate that CH is more resistive than Al at lower temperatures, although this is not true above 20 eV. Most of the CH target reaches temperatures above 20 eV and consequently, a high degree of filamentation is not expected in the CH target at these early time-scales, consistent with our experimental observations.

Figure 4a shows the background electron temperature contour plot in the CH target along the longitudinal *x* − *y* plane, 0.7 ps after the interaction. The saturated area in the figure close to the interaction represents regions with temperatures greater than 100 eV. As can be seen, the temperature remains above 20 eV throughout most of the hot electron distribution. At these temperatures, the resistivity of CH can be lower than Al. Figure 4b shows the resistivity of CH (green) and Al (red) as a function of temperature, using the Lee-More resistivity model^36^. Although CH is more resistive than Al at lower temperatures, this is not so above 20 eV. As the temperature remains above 20 eV throughout most of the hot electron distribution in the CH target soon after the laser irradiation, a high degree of filamentation is not expected at early time-scales, consistent with our experimental observations. At later time-scales, the hot electron distribution and consequently the magnetic field profile suffer from filamentation, owing to a fall in the temperature, which may be further affected by the dynamics of the evolving sheath field structure at the target rear^12, 27, 28^.

Recent experiments by Scott *et al*.^27^ demonstrated the effect of Weibel instability, evolving as a function of the scale-length of the rear-side plasma. This was experimentally achieved by a controlled, time-delayed prepulse incident on 45-*μ*m thick graphite foils and 5- and 25-*μ*m thick Au foils at an intensity of 4 × 10^18^ W/cm^2^, the evolution of the Weibel instability being imprinted on the accelerated proton beam at the target rear particularly at time-scales of several tens of picoseconds. Similar results have also been recently reported by Göde *et al*.^28^, where strong spatial modulations of the proton beam profile were observed for a micron-scale solid-density hydrogen jet at an intensity of 5 × 10^20^ W/cm^2^, attributed to Weibel-like instabilities in the rear-side plasma, particularly near the critical density. In light of the above experiments, it is plausible that Weibel-like filamentary instabilities in the micron-scale rear-surface plasma may play a pivotal role in inducing the filamentary structures we observe in the rear-surface magnetic field profile at later time-scales of ~10 ps, in addition to the resistive filamentation^1^ induced in the bulk of the target. Such filamentary instabilities hosted in the rear-surface sheath are not prominent at earlier time-scales of a few picoseconds, as observed in our magnetic field measurements at initial time-scales, and consistent with measurements by Scott *et al*.^27^.

In conclusion, we have explored the magnetic fields at the rear of solid targets, generated by hot electrons originating from the intense laser-solid interaction at the target front surface. The optical polarimetry we employed is a sensitive technique that enables us to resolve the dynamics of hot electron propagation with high spatial and temporal resolution. As a result, we infer snapshots of the hot electron distribution through a CH target at different time-scales and identify an interaction regime in terms of local temperature where the transport can be smooth and beam-like. The hot electron distribution not only depends on the initial conductivities of the materials but also on how the conductivity changes with temperature. In fact, the local temperature or resistivity inside the solid is inherently transient and is expected to be a complex function of the distance from the interaction point, local lattice configurations^37^ and laser parameters like intensity, pulsewidth and contrast^38^. It is therefore essential to have a diagnostic that can unravel the complex dynamics of hot electron propagation through solids in order to optimise it. This is of critical importance in developing novel sources for energetic ions and engineering innovative techniques for long-range energy transport^39^. The experimental snapshots presented here highlight the complexity in the phenomenon and suggest that it is highly transient in nature, yet amenable to accurate and detailed measurement. This measurement technique would enable us to extend these studies to obtain a full spatio-temporal understanding and a potential control of the hot electron transport process that is so central in intense-laser-plasma research; further investigations to that end are under way.

## Methods

### Experimental setup

The *p*-polarized interaction (pump) laser pulse was focused on the target by an *f*/3 off-axis parabolic mirror at an angle of incidence of 30°. Measurements at low intensities using a microscope objective normal to the target plane estimated the focal spot to be 4 *μ*m (FWHM), containing about 30% of the laser energy in the focal volume, resulting in an estimated peak intensity of ~4 × 10^20^ W/cm^2^. The amplified spontaneous emission (ASE) contrast was measured to be 10^10^ at 1 ns and the contrast was better than 10^8^ at 100 ps.

The target surface was examined with a white-light interferometer, with a resolution <50 nm, and no micron-scale initial granularity of the target surface was observed.

A linearly-polarized, time-delayed and frequency-doubled (*λ* = 526 nm) probe pulse, extracted from the main interaction pulse, was focused to a 50-*μ*m diameter spot on the target rear at near-normal incidence (~3°). The probe energy was suitably attenuated to a few hundred mJ to allow detection of the probe above the plasma emission as well as transition radiation in the charge-coupled-devices (CCDs), while ensuring that the probe is non-intrusive and non-perturbative with regard to the magnetic field measurement (as indicated by Fig. 2a).

The magnetic fields induce a birefringence in the plasma at the target rear, resulting in a change in the polarization state of the incident probe. Briefly, the phase difference between the ordinary *O*-wave and the extra-ordinary *X*-wave of the external probe, reflected from the critical surface and the *X*-wave cutoff respectively, induces an ellipticity in the probe, which can be expressed in terms of the differences between the refractive indices of the *O*- and *X*-waves in accordance with the Appleton-Hartree formula. Since the refractive index of the *X*-wave depends on the ambient magnetic field via the cyclotron frequency, the ellipticity induced in the probe can be uniquely mapped on to the magnetic field experienced by the probe^24, 25^.

An optical streak camera was employed to synchronize the probe pulse with the main interaction pulse, the synchronization being limited by the pulsewidth of 2.5 ps. All temporal delays between the probe and the main interaction pulse mentioned are peak-to-peak measurements of the streak camera. A complete characterization of the polarization of the reflected probe was performed by measuring all the Stokes’ parameters (*s*_1_, *s*_2_, *s*_3_) of the reflected probe, using high-extinction-ratio polarizers, quarter-wave-plates and CCDs, coupled with interference filters. In particular, a polarizer aligned parallel to the incident polarization gave ${I}_{1}={I}_{0}\mathrm{(1}+{s}_{1}\mathrm{)/2}$, whereas a polarizer aligned at an angle of 45° to the incident polarization gave ${I}_{2}={I}_{0}\mathrm{(1}+{s}_{2}\mathrm{)/2}$, where *I*_0_ is the intensity of the reflected probe. In addition, a quarter wave-plate, along with a polarizer aligned at an angle of 45° with respect to the quarter-wave-plate, gave ${I}_{3}={I}_{0}\mathrm{(1}+{s}_{3})/2$. The Faraday rotation *ψ* and the induced ellipticity *χ* were then measured from the Stokes’ parameters since ${s}_{1}={\rm{c}}{\rm{o}}{\rm{s}}\,2\chi {\rm{c}}{\rm{o}}{\rm{s}}\,2\psi$, ${s}_{2}={\rm{c}}{\rm{o}}{\rm{s}}\,2\chi {\rm{s}}{\rm{i}}{\rm{n}}\,2\psi$ and ${s}_{3}={\rm{s}}{\rm{i}}{\rm{n}}\,2\chi$ ^25, 40^.

The amplitude of the magnetic fields at the target rear depends on the scale-length of the rear-side plasma. Consequently, 1D radiation hydrodynamics simulations using the HYADES code^41^ were performed to obtain the scale-length of the plasma density profile at the target rear, assuming that the target rear was volume-heated to a temperature consistent with that observed for similar targets in previous experiments under similar conditions^42, 43^. These simulations were run for a CH target for the time-period (1-10) ps following the main interaction pulse and the expansion velocity of the critical surface was found to be fairly constant at 9 × 10^6^ cm/s during the simulation period equivalent to a scale-length of <1 *μ*m at 10 ps at the target rear, consistent with shadowgraphy measurements). The exponential scale-lengths were obtained from the plasma density profiles near the critical density of the reflected probe. The error bar in the temperature was taken into account while calculating the error bar in the magnetic field. The fact that a 100 eV change in the temperature resulted in only 1-2 MG change in the magnetic field indicates that the magnetic field magnitude is not too sensitive to the scale-length of the rear-side plasma for our experimental conditions and consequently, an approximate target-rear expansion velocity calculated by the 1D radiation hydrodynamics simulation suffices.

### Simulation details

ZEPHYROS is a 3D hybrid code, where the hot electron population is treated as macro-particles, while the background electrons and ions are treated as a two-temperature fluid. A detailed description of the methods and approximations may be found in refs 44–46. The generation and evolution of the magnetic field **B** is represented by the well-known equation^45^:

$$\frac{\partial {\bf{B}}}{\partial t}=\eta \nabla \times {{\bf{j}}}_{f}+\nabla \eta \times {{\bf{j}}}_{f}+\frac{\eta }{{\mu }_{o}}{\nabla }^{2}{\bf{B}}-\frac{1}{{\mu }_{0}}\nabla \eta \times {\bf{B}},$$

where *η* is the local resistivity (a function of the background temperature), **j**_*f*_ is the fast electron current density and *μ*_0_ is the permeability of free space.

The ZEPHYROS simulations were performed using a 100 × 280 × 280 box with a cell size of 0.5 × 0.5 × 0.5 *μ*m^3^ up to 0.7 ps. The electrons were injected from a region in the center of the *x* = 0 plane so as to model laser irradiation at 4 × 10^20^ W/cm^2^. A laser-to-hot-electron conversion efficiency of 30% was assumed^47^. The transverse ‘laser spot’ profile was chosen to be a Gaussian function with an FWHM of 4 *μ*m. The hot electron energy distribution used was an exponential distribution ($\propto {\rm{e}}{\rm{x}}{\rm{p}}(-\varepsilon /\bar{\varepsilon })$) with the mean energy, $\bar{\varepsilon }$, determined by the Wilks’ ponderomotive scaling^48^. The angular distribution of the hot electrons was considered to be $\propto {{\rm{c}}{\rm{o}}{\rm{s}}}^{2}\theta$, where $\theta$ is the divergence angle. The background material used was CH at an initial temperature of ~1 eV. The resistivity of CH was determined using the Lee-More model^36^, which was found to be most appropriate for our experimental conditions^49^. The *x*-boundaries of the simulation box were reflective to allow refluxing^50^, but the transverse boundaries were open.

### Data Availability

Data associated with research published in this paper can be accessed at http://dx.doi.org/10.5286/edata/706.
